# On the Stability of Uranium Carbide in Aqueous Solution—Effects of HCO_3_^–^ and H_2_O_2_

**DOI:** 10.1021/acsomega.1c04581

**Published:** 2021-09-10

**Authors:** Sawsan El Jamal, Mats Johnsson, Mats Jonsson

**Affiliations:** † School of Engineering Sciences in Chemistry, Biotechnology and Health, Department of Chemistry, KTH Royal Institute of Technology, Stockholm SE-100 44, Sweden; ‡ Department of Materials and Environmental Chemistry, Stockholm University, Stockholm SE-106 91, Sweden

## Abstract

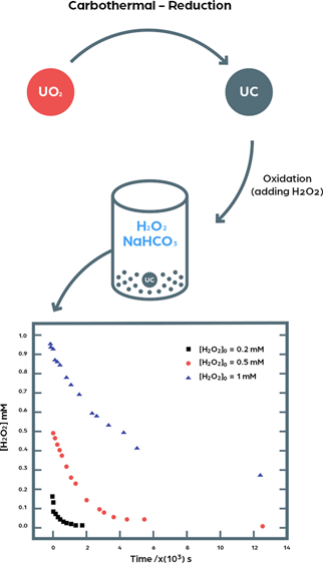

Uranium carbide (UC) is a candidate fuel material for future Generation IV nuclear reactors. As part of a general safety assessment, it is important to understand how fuel materials behave in aqueous systems in the event of accidents or upon complete barrier failure in a geological repository for spent nuclear fuel. As irradiated nuclear fuel is radioactive, it is important to consider radiolysis of water as a process where strongly oxidizing species can be produced. These species may display high reactivity toward the fuel itself and thereby influence its integrity. The most important radiolytic oxidant under repository conditions has been shown to be H_2_O_2_. In this work, we have studied the dissolution of uranium upon exposure of UC powder to aqueous solutions containing HCO_3_^–^ and H_2_O_2_, separately and in combination. The experiments show that UC dissolves quite readily in aqueous solution containing 10 mM HCO_3_^–^ and that the presence of H_2_O_2_ increases the dissolution further. UC also dissolves in pure water after the addition of H_2_O_2_, but more slowly than in solutions containing both HCO_3_^–^ and H_2_O_2_. The experimental results are discussed in view of possible mechanisms.

## Introduction

Future-generation IV nuclear reactor systems will be significantly more sustainable, and the reactors are designed to be safer than their predecessors.^1,2^ There are currently several reactor concepts under development and, in parallel with this, several fuel materials are being investigated.^3−6^ Generation IV reactors will be used in combination with reprocessing plants for the used nuclear fuel which will limit the amount of long-term radioactive waste to be placed in geological repositories.^7^ Nevertheless, the possibility that the novel fuel materials have to be placed in deep geological repositories, for example, due to sudden changes in policies, should be considered. The behavior of conventional UO_2_-based spent nuclear fuel under repository conditions has been studied quite extensively for almost half a century.^8,9^ The worst case scenario in the safety analysis of a geological repository for spent nuclear fuel involves a complete barrier failure where groundwater can come in contact with the fuel. This enables the dissolution of the fuel matrix and subsequent release of radionuclides into the groundwater.^10,11^ In the case of UO_2_, the fuel matrix has very low solubility under the reducing groundwater conditions that often prevail at locations that are deemed suitable for deep geological repositories.^12,13^ The inherent radioactivity of the spent nuclear fuel will cause radiolysis of the groundwater and thereby contribute to the production of oxidants (HO^•^, HO_2_^•^, and H_2_O_2_) and reductants (e_aq_^–^, H^•^ and H_2_).^14^ For kinetic reasons, the oxidants will dominate the chemistry of the fuel surface and convert U(IV) to significantly more soluble U(VI) and thereby cause oxidative dissolution of the fuel matrix.^8,9,15^ Under a typical canister failure scenario, oxidant production at the fuel surface will be dominated by α-radiolysis and fuel oxidation can be almost exclusively attributed to H_2_O_2_.^8,15^ The commonly occurring groundwater constituent HCO_3_^–^ facilitates this process since U(VI) forms stable and soluble carbonate complexes.^12,16^ Several studies have been conducted in order to understand the mechanism and kinetics of the reaction between H_2_O_2_ and UO_2_-based materials.^15−21^

Uranium carbide (UC) has received some attention as a potential nuclear fuel for generation IV reactors.^6^ Its thermal conductivity is 20 W m^–1^ K^–1^^22^ which is 10 times higher than for the oxide.^23^ The carbide also has smaller thermal expansion. Therefore, it will be possible to operate reactors at higher temperature and the fuel pin design will be more flexible. Another important factor is the high fissile material density of UC compared to standard UO_2_-based fuels.^24^

It is known that metal carbides undergo hydrolysis.^25^ Depending on the nature of the metal, specific gaseous hydrolysis products are formed. For carbides of the alkaline-earth metal group except for beryllium, acetylene or methyl acetylene gas is formed.^26^ For carbides of the transition metal group IV and V, methane is formed.^25^ Similarly to the other metal carbides, uranium carbide undergoes hydrolysis. Hydrolysis of uranium carbide has been studied quite extensively under acidic conditions for the purpose of developing nuclear fuel reprocessing.^27−29^ UC exposed to water at neutral pH between 25 and 99 °C has been shown to yield hydrous uranium oxide, hydrogen gas, and a complex mixture of gaseous hydrocarbons, mainly methane with small amount of ethane and saturated C3- to C6- hydrocarbons. The hydrolysis reaction is often generally described as
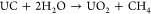


The temperature affects the rate of hydrolysis making it faster at high temperature; however, it does not cause any change concerning the composition of the hydrolysis gaseous products.^30^ In alkaline solutions, hydrolysis of UC has been shown to yield U(VI).^31^ Hydrolysis connected to an increase in the oxidation number for the metal has also been suggested for WC, where WO_3_ is formed upon hydrolysis.^32^

While the hydrolysis of uranium carbide has been studied quite extensively, studies of oxidative dissolution of uranium carbide under conditions similar to those expected in a geological repository for spent nuclear fuel cannot be found in the literature. However, there are studies of the behavior of spent HTR (high temperature reactor) fuel under simulated repository conditions. HTR fuel has a complex structure of small UO_2_ particles (kernel) covered with layers of pyrolytic carbon and SiC constituting a fuel particle.^33^ Of particular interest, there is the study of SiC corrosion. SiC corrosion experiments were performed in solutions mimicking granitic groundwater and clay pore water at different pH. The solutions were exposed to air, and the rate of corrosion was found to increase with increasing pH. It was also quite clear that the rate of corrosion decreases with time which was attributed to the formation of an oxide layer covering the SiC surface.^33^

In this work, we have explored the stability of UC in aqueous solution with particular focus on the effects of HCO_3_^–^ as the groundwater constituent and H_2_O_2_ as the dominant aqueous radiolysis product.

## Results and Discussion

**Figure 1:**
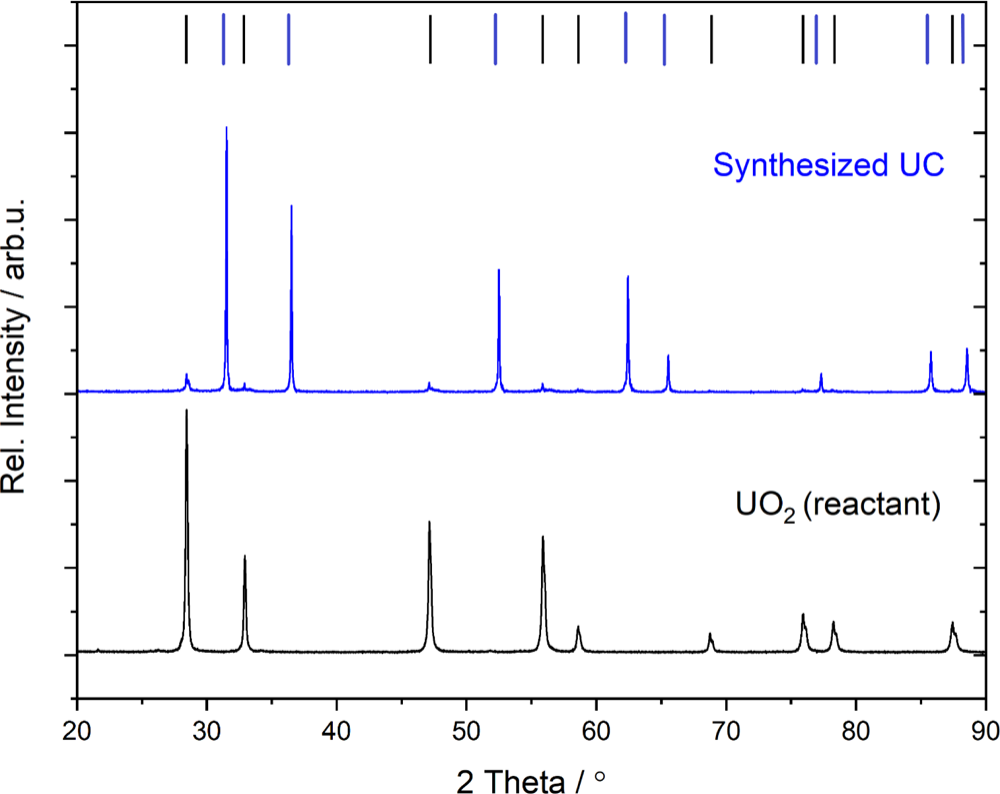
XRD pattern of the reactant UO_2_ (black) and the synthesized UC (blue). Indices (blue) for UC from ref (34), ICSD collection code [26476]. Indices (black) for UO_2_ from ref (35), ICSD collection code [24850].

The synthesized UC powder was analyzed using XRD. The diffractogram is shown in Figure 1 along with a diffractogram for UO_2_. As can be seen, the main product of the synthesis is indeed UC. However, there are still traces of the unreacted UO_2_. The amount of the remaining UO_2_ was estimated to be 7% in the UC product. This should be kept in mind since these traces of UO_2_ might affect the reactivity of the UC.

### UC Stability in Aqueous Solutions Continuously Purged with N_2_

**Figure 2:**
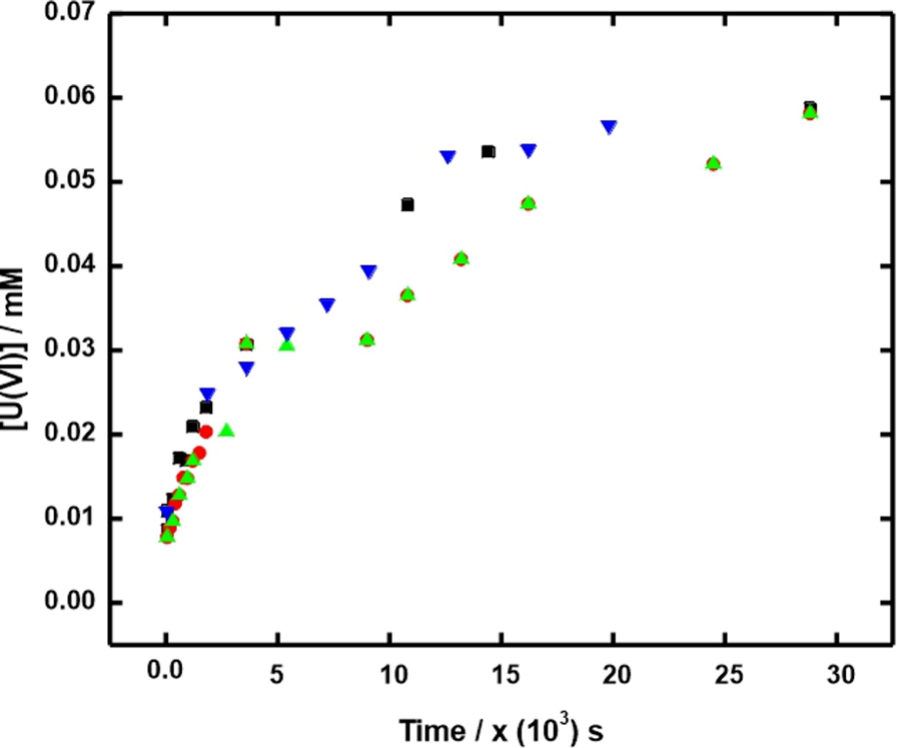
Uranium dissolution for 30 mg UC in 25 mL aqueous solution of 10 mM NaHCO_3_ purged with N_2_ as a function of time. Four parallel experiments of the same duration were performed, each set represented by a separate symbol.

To quantify oxidative dissolution, the dissolution in the absence of added oxidants must first be known. The stability of UC in aqueous solutions continuously purged with N_2_ to remove dissolved O_2_ was studied by measuring the uranium concentration as a function of time in pure water and in an aqueous solution containing 10 mM HCO_3_^–^. The suspensions had a total volume of 25 mL. For the experiment performed in pure water, 80 mg of UC was used to prepare the suspension while for the experiment performed in 10 mM HCO_3_^–^ solutions, 30 mg of UC was used. This difference in the amount of powder is mainly due to the fact that the dissolution is faster in a bicarbonate containing solution than in pure water. The experiment performed in pure water did not reveal any dissolved uranium U(VI) above the detection limit after 8 h. The detection limit determined for the U(VI) species resulting from the dissolution of UO_2_ using the Arsenazo III method is 5 × 10^–7^ M.^19^ The results for the experiments performed in 10 mM HCO_3_^–^ are presented in Figure 2.

As can be seen, there is a significant dissolution of uranium in the presence of HCO_3_^–^. This implies that soluble U(VI) is formed in the process in analogy with what has previously been reported for hydrolysis of UC in alkaline solutions.^31^

### UC Reactivity toward H_2_O_2_

**Figure 3:**
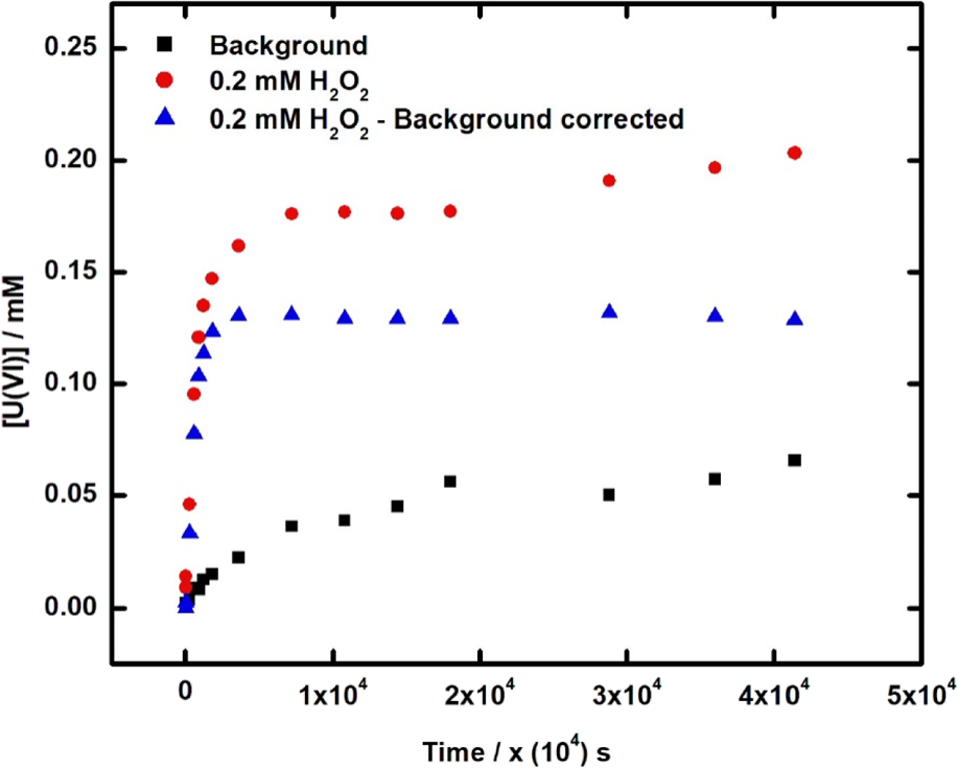
Uranium dissolution for 30 mg UC in a 25 mL of 10 mM NaHCO_3_ in the absence and presence of 0.2 mM H_2_O_2_ with and without the background correction.

**Figure 4:**
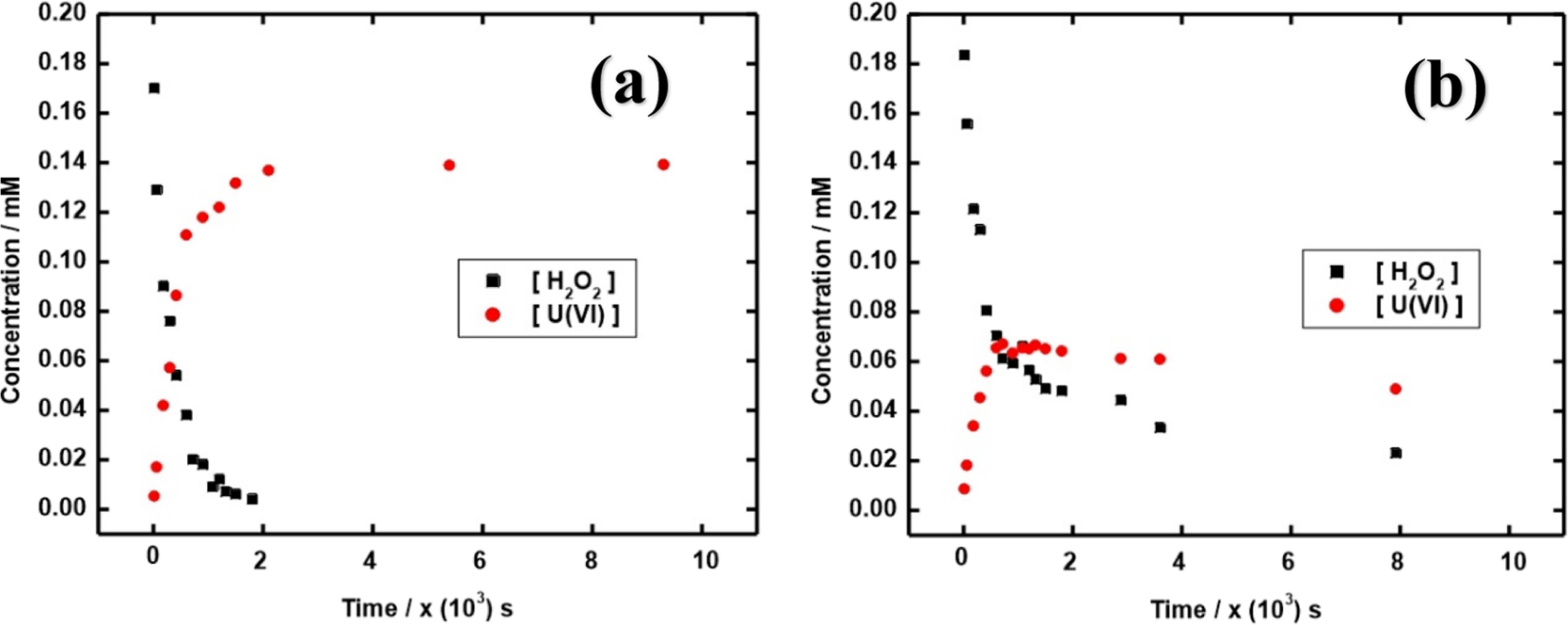
H_2_O_2_ consumption and uranium dissolution as a function of time for 30 mg UC in aqueous solutions: (a) 10 mM NaHCO_3_ and (b) 0 mM NaHCO_3_.

H_2_O_2_ has been shown to be the radiolytic oxidant that has the highest impact in radiation-induced oxidative dissolution of UO_2_-based spent nuclear fuel under deep repository conditions.^15^ For this reason, it is important that every new fuel matrix is characterized in terms of its reactivity toward H_2_O_2_. In Figure 3 the concentrations of uranium as function of time for UC in 10 mM NaHCO_3_ in the presence and absence of 0.2 mM H_2_O_2_ are shown. The solutions were continuously purged with N_2_ to remove dissolved O_2_ and thereby preventing it from contributing to the process. As can be seen, considerably more uranium is dissolved in the presence of H_2_O_2_. The difference between the two sets of data presents the corrected amount of dissolved uranium attributed to the oxidation of UC by H_2_O_2_. In Figure 4, the H_2_O_2_ and U(VI) concentrations with background correction are plotted as function of exposure time in an aqueous solution containing 10 mM HCO_3_^–^ and in a solution with no added HCO_3_^–^.

As can be seen, the H_2_O_2_ concentration decreases quite rapidly in both cases. However, there are clear differences between the two systems. In the HCO_3_^–^ containing system, H_2_O_2_ is completely consumed and uranium is dissolved to a much larger extent than in the system without added HCO_3_^–^. In the latter system, uranium is initially dissolved quite rapidly, but the concentration reaches a maximum, whereafter it slowly decreases. The H_2_O_2_ concentration in the same system decreases quite slowly after the initial rapid decrease. The evolution of the H_2_O_2_ and uranium concentrations in the HCO_3_^–^ deficient system can probably be attributed to the formation of a secondary phase, probably studtite [UO_2_(O_2_) (H_2_O)_4_].^36−38^ The same reaction dynamics, that is, that the uranium concentration initially increases rapidly and thereafter slowly decreases has been observed for UO_2_ as well in solutions without added HCO_3_^–^. The slow precipitation of studtite from fairly colloidally stable studtite particles makes it difficult to experimentally determine the concentrations of free H_2_O_2_ and U(VI) in these solutions.^36^

To further study the kinetics of the reaction between H_2_O_2_ and UC, the same type of experiment was performed for various amounts of UC (15, 25, 30, 60, and 80 mg) in 25 mL 10 mM bicarbonate solution and in pure water. Under these conditions, the consumption of H_2_O_2_ was found to follow first-order kinetics during the initial phase of the reaction. At higher conversion, the formation of colloidally stable studtite particles (pure water)^36^ and uranyl-peroxo-carbonate complexes^39−41^ start to influence the analysis as well as the reaction dynamics of the system. The resulting first-order rate constants are shown in Table1.

**Table 1:** First-Order Rate Constant for Different Amounts of UC Over Time in a 25 mL Aqueous Solutions: 10 mM HCO_3_^–^ Solution and Water, [H_2_O_2_]_0_ = 0.2 mM

mass (mg) of UC	*k* (s^–1^) in 10 mM HCO_3_^–^	*k* (s^–1^) in 0 mM HCO_3_^–^
15	1.3 × 10^–3^	8.0 × 10^–4^
25	1.9 × 10^–3^	1.5 × 10^–3^
30	2.4 × 10^–3^	1.6 × 10^–3^
60	4.3 × 10^–3^	2.1 × 10^–3^
80	5.6 × 10^–3^	3.2 × 10^–3^

**Figure 5:**
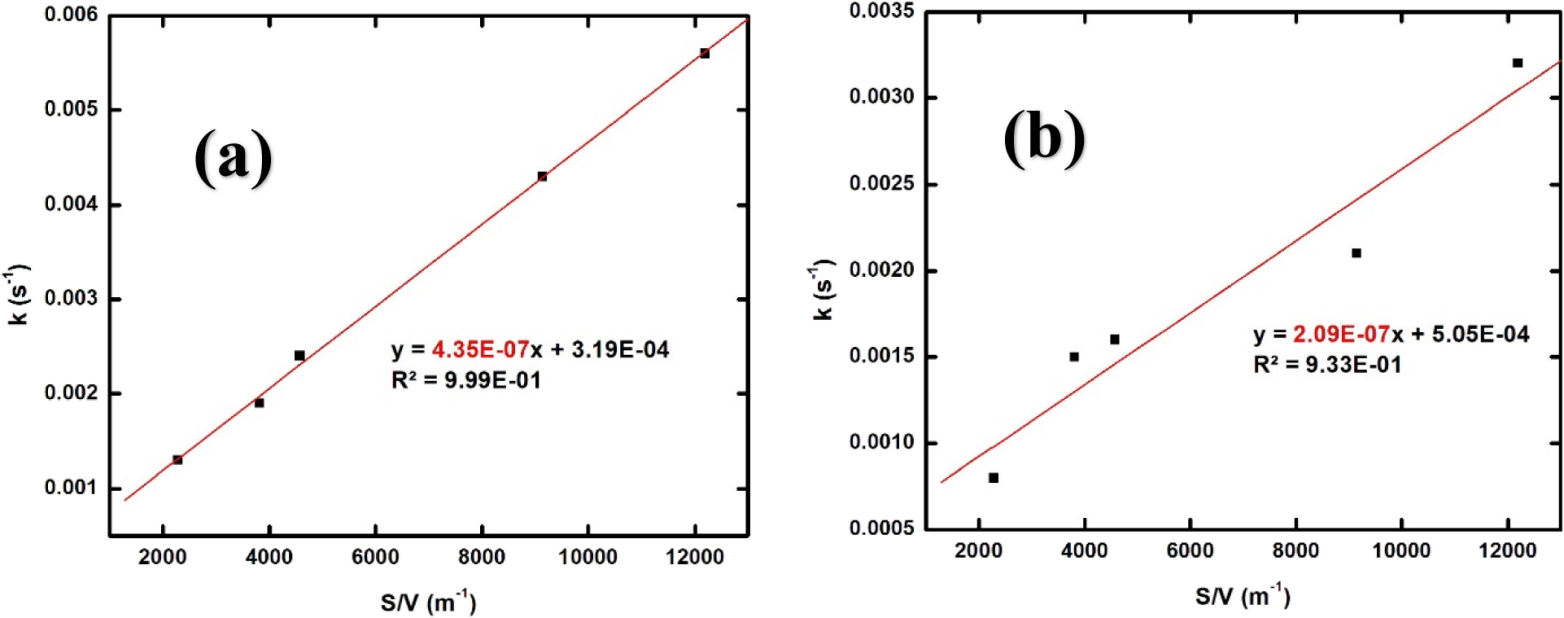
First-order rate constant *k* (s^–1^) as a function of solid surface area over total solution volume ratio [*S*/*V* (m^–1^)] for different amounts of UC (15, 25, 30, 60, and 80 mg) in 25 mL aqueous solutions: (a) 10 mM NaHCO_3_ and (b) 0 mM NaHCO_3_.

As can be seen, the first-order rate constant increases with increasing amount of UC (i.e., with increasing UC surface area to solution volume ratio). In general, the difference between the two systems is slightly less than a factor of two in favor of the HCO_3_^–^ containing system. Based on the first-order rate constants, we can derive the heterogeneous second-order rate constants for the reaction between H_2_O_2_ and UC by plotting the first-order rate constants toward the UC solid surface area to solution volume ratio. The slope of such plot yields the heterogeneous second-order rate constant in the unit m s^–1^. The plots are shown in Figure 5.

As can be seen in Figure 5, the second-order rate constants are 4.35 × 10^–7^ and 2.09 × 10^–7^ m s^–1^ in 10 mM bicarbonate solution and pure water, respectively. The second-order rate constants based on the initial consumption of H_2_O_2_ differ by a factor of two between the two systems. It should be noted that the linear correlation is significantly poorer for the system without HCO_3_^–^. The intercepts are 3.19 × 10^–4^ and 5.05 × 10^–4^ s^–1^. Given the uncertainty in the intercept for the system without HCO_3_^–^ (±2 × 10^–4^ s^–1^), the two intercepts are not significantly different. The intercept could in general be attributed to other reactions consuming H_2_O_2_ such as reactions with the surfaces of the reaction vessel or impurities in solution.

The reactivity of UC toward H_2_O_2_ is higher (by ca. 30%) than the reactivity of U_3_Si_2_, an accident tolerant fuel candidate that was recently studied.^38^ U_3_Si_2_, in turn, was found to be around one order of magnitude more reactive than UO_2_.^38^ In other words, the reactivity of UC toward H_2_O_2_ is higher than that of UO_2_ and U_3_Si_2_. For UC, it should be kept in mind that extensive hydrolysis would result in formation of a UO_2_ surface and the reactivity would then not be expected to differ from that of UO_2_. The fact that there is still a difference in reactivity implies that the hydrolysis is not sufficiently fast to quantitatively convert the UC surface to UO_2_ under the present conditions. In terms of uranium dissolution, UC, U_3_Si_2_, and UO_2_ display similar behavior regarding the effect of HCO_3_^–^ as compared to solutions without added HCO_3_^–^. In general, the amount of dissolved uranium is higher in the presence of HCO_3_^–^ than in pure water. In 10 mM HCO_3_^–^, the uranium concentration increases as long as there is still H_2_O_2_ in solution. However, in solutions without added HCO_3_^–^, the uranium concentration increases until reaching a maximum value, whereafter it slowly decreases, even though the solution still appears to contain H_2_O_2_. As mentioned above, this can be attributed to the formation of studtite.

**Figure 6:**
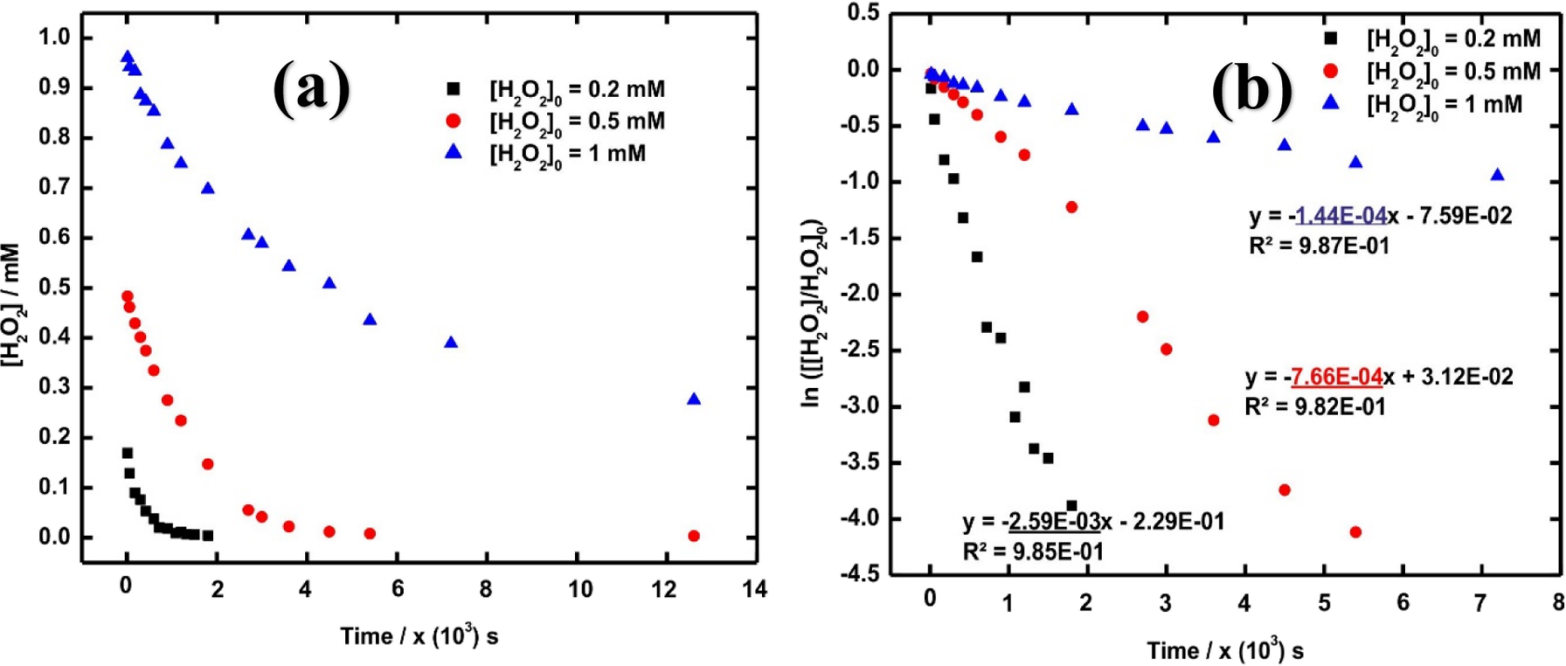
(a) H_2_O_2_ concentration as a function of reaction time for different initial concentrations (0.2, 0.5, and 1 mM) in a suspension of 30 mg UC in 25 mL 10 mM NaHCO_3_ and (b) logarithm of the H_2_O_2_ concentration as a function of reaction time for the same systems as in (a).

To investigate the effect of H_2_O_2_ concentration on the kinetics of H_2_O_2_ consumption on UC, we monitored the H_2_O_2_ concentration as a function of reaction time in suspensions containing 30 mg UC powder in 25 mL of 10 mM HCO_3_^–^ solution for three different initial H_2_O_2_ concentrations. The results are shown in Figure 6 together with the corresponding first-order plots. From Figure 6b, it is evident that H_2_O_2_ consumption follows first-order kinetics at all three initial H_2_O_2_ concentrations. However, the first-order rate constant displays concentration dependence. The only possible rationale for this is that the reaction mechanism changes with increasing initial H_2_O_2_ concentration. At low initial H_2_O_2_ concentration, the kinetics for H_2_O_2_ consumption is most probably governed by adsorption of H_2_O_2_ to the UC surface, while at higher initial H_2_O_2_ concentrations other reactions, possibly involving already adsorbed H_2_O_2_, become rate-limiting. This also depends on the relative rates of oxidation and hydrolysis since the latter will alter the composition and thereby the reactivity of the surface.

To shed some more light on the effect of initial H_2_O_2_ concentration, we can calculate the so-called dissolution yield. The dissolution yield is the ratio between the amount of dissolved uranium and the amount of consumed H_2_O_2._ The dissolution yield has been used quite extensively to assess the competition between oxidation of UO_2_ and the catalytic decomposition of H_2_O_2_ on UO_2_.^20,42^ To determine the dissolution yields in the present system, different initial concentrations of H_2_O_2_ were added to 25 ml aqueous suspensions of 30 mg UC in 10 mM NaHCO_3_. The uranium concentrations were measured after the H_2_O_2_ was completely consumed. In previous studies on UO_2_, the dissolution yield has been shown to depend on the initial H_2_O_2_ concentration.^43^ It was shown that the dissolution yield decreases with the increase of the initial H_2_O_2_ concentration. In this case, the concentration dependence was attributed to the reaction mechanism. It has been shown that H_2_O_2_ can react with UO_2_ both via oxidation of the surface and via catalytic decomposition of H_2_O_2_ on the surface. The latter reaction consumes H_2_O_2_ but does not lead to uranium dissolution. Both reactions proceed via a common intermediate, a surface-bound hydroxyl radical.^43^ In the catalytic decomposition of H_2_O_2_, the surface-bound hydroxyl radical (formed upon adsorption of H_2_O_2_) reacts with H_2_O_2_ to produce H_2_O and HO_2_^•^. This reaction competes with the electron-transfer from U(IV) to the surface-bound hydroxyl radical subsequently leading to oxidative dissolution. As a consequence, the dissolution yield decreases with increasing H_2_O_2_ concentration. For the UC system, the results are shown in Table 2.

**Table 2:** Dissolution Yield of Uranium (VI) for Different Initial H_2_O_2_ Concentrations in 25 mL 10 mM HCO_3_^–^Aqueous Solutions Containing 30 mg UC

[H_2_O_2_] mM	[U] mM	dissolution yield (%)
0.1	0.073	73.2
0.2	0.133	66.7
0.5	0.251	50.3
1	0.491	49.1

As for the UO_2_ system, the dissolution yield decreases with increasing initial H_2_O_2_ concentration. However, it is quite clear that the maximum dissolution yield for UC is much lower than for UO_2_ under comparable conditions yet higher than for U_3_Si_2_.^38^ This can partly be attributed to the fact that oxidative dissolution of UC is not necessarily a strict two-electron process which was also demonstrated for U_3_Si_2_, where H_2_O_2_ is also consumed by oxidizing Si.^38^ Hence, H_2_O_2_ may also be consumed in oxidation of carbon.

The change in dissolution yield with increasing initial H_2_O_2_ concentration is somewhat unexpected for UC since catalytic decomposition of H_2_O_2_ is not expected to occur as a competing process. As it is quite probable that extended hydrolysis will lead to formation of more UO_2_ on the UC surface, the surface reactivity could change to that of UO_2_ which would enable catalytic decomposition of H_2_O_2_. To test this hypothesis, the effect of repeated exposure to H_2_O_2_ has been studied. As presented in Supporting Information, 30 mg of UC powder in a 25 mL volume of 10 mM HCO_3_^–^ solution was exposed three consecutive times to 0.2 mM H_2_O_2_. The concentration of H_2_O_2_ was monitored as function of time, and a very slight change (decrease) in reactivity was observed between the three consecutive exposures. As for the dissolved uranium, these multiple exposures led to the release of the same amount of U(VI) in every exposure. No significant changes in the rate of dissolution were observed. As mentioned above, UO_2_ is slightly less reactive toward H_2_O_2_ than UC. The slight decrease in reactivity toward H_2_O_2_ observed as a consequence of consecutive exposures may therefore be attributed to the formation of UO_2_ on the UC surface due to the extended time for hydrolysis.

Given the considerably lower oxidant concentrations in a geological repository as compared to the experiments performed here, it is very likely that surface hydrolysis will be a faster process relative to surface oxidation. This would imply that the reactive fuel surface of UC would essentially be UO_2_ and the behavior of spent UC-based fuel would not differ from that of spent UO_2_-based fuel under geological repository conditions.

## Conclusions

The experiments performed in this work show that the aqueous radiolysis product H_2_O_2_ reacts with the potential nuclear fuel material UC resulting in oxidative dissolution of the latter. The reaction is faster in aqueous solutions containing HCO_3_^–^ than in pure water which implies that HCO_3_^–^ facilitates dissolution of oxidized UC through the formation of soluble complexes. UC is found to be more reactive toward H_2_O_2_ than is the conventional nuclear fuel material UO_2_. However, consecutive exposures to H_2_O_2_ appears to convert the UC surface to UO_2_ as reflected by a slight change in reactivity. It is clear that the dissolution yield (expressed as amount of uranium released per consumed H_2_O_2_) is lower for UC than for UO_2_ under the same conditions. However, the fact that the trend in dissolution yield as a function of initial H_2_O_2_ concentrations largely parallels that of UO_2_ also highlights the importance of the change in surface composition.

## Experimental Section

### Material Preparation and Characterization

UC was prepared by carbo-thermal reduction of a mixture of UO_2.0_ and carbon powder with a molar ratio of 1 to 3.^44^ The powdered mixture was wet-milled in 2-propanol, dried in an argon atmosphere, and pressed into small pellets. During the carbo-thermal reduction,^45^ the pellets were heated under argon (>99.999%, Strandmöllen) at a temperature of 1500 °C for 10 h to form UC. The reduced pellets were crushed. XRD measurements confirmed that the synthesized powder is UC. The specific surface area of the crushed UC is 3.81 m^2^/g. The depleted uranium dioxide powder was supplied by Westinghouse Electric Sweden AB. This UO_2_ is hyper-stoichiometric; it was therefore reduced under 5% H_2_ in N_2_ (Strandmöllen) at 450 °C for 9 h to form the UO_2.0_ used in the carbo-thermal reduction. The carbon black powder (≥99.9%, acetylene, 50% compressed) was supplied by Alfa Aesar.

### Powder X-Ray Diffraction

A PANalytical XPert PRO diffractometer using Cu Kα radiation in a 2θ range between 5 and 90^°^ at room temperature was used to run XRD measurements for the reactant and the synthesized UC powder.

### BET Surface Area

BET surface area of the UC powder was measured on a Micromeritics 3Flex 3500. The specific surface area for the UC powder is 3.81 m^2^/g according to BET measurements.

### Effect of H_2_O_2_ on Uranium Carbide Dissolution Kinetics in Aqueous Solutions

The synthesized uranium carbide powder was stored in the glovebox under argon atmosphere. Aqueous powder suspensions (25 mL) of UC were prepared using MilliQ water (18.2 M ohm cm, Merck Milli-Q) containing 10 mM NaHCO_3_ (Merck) or no added NaHCO_3_. To remove the excessive carbon that resulted from the synthesis, the UC powder was washed four times in solutions of the same composition as in the subsequent experiment. After washing the powder, the aqueous powder suspensions were prepared by adding to the UC powder the adequate solution whether it is water containing 10 mM NaHCO_3_ or pure water. The pH measured initially in both of these suspensions was 8.9 and 7.6, respectively, for water containing bicarbonate and pure water. Once the pH was measured, the suspensions were stirred and purged with N_2_ (>99.999%, Strandmöllen) throughout the experiment at 20 °C. The purging with N_2_ was done to remove dissolved oxygen and to prevent oxygen intrusion. In all experiments, the reaction vessels were protected from light in order to prevent influence from photolysis. 8 h after preparing the UC suspensions, the pH had increased to 9.1 in pure water, while it remained constant in the bicarbonate-containing solutions. Concentrations of U(VI) were measured over time throughout the experiment using the Arsenazo III method.^46^ This method is based on UV–vis spectroscopic quantification of the complex between uranium (VI) and the reagent Arsenazo III (1,8 dihydroxynaphtalene-3,6-disulphonic acid-2,7-*bis*[(azo-2)-phenylarsonic acid]). The complex has a maximum absorbance at 653 nm. In order to measure the concentration of U(VI), a volume ranging between 50 and 400 μL needs to be extracted from the aqueous-powdered UC suspension. At the beginning of the reaction, the concentration of dissolved uranium U(VI) is low; therefore, a relatively large volume is needed to measure it. Since over time, the concentration of U(VI) increases and lower extracted volume from the aqueous suspension will be needed to actually measure its concentration. Depending on the sample volume extracted from the aqueous UC powder suspension, Milli-Q water will be added to a total volume of 1.5 mL. To this 1.5 mL volume of U(VI) and Milli-Q water, two different reagents are added. These two reagents consist of 40 μL of 0.16%(wt) of Arsenazo III and 60 μL of 1 M HCl so that the total volume analyzed to determine the U(VI) concentration is 1.6 mL.^46,47^

In experiments exploring the effect of H_2_O_2_, UC powder suspensions contained an initial concentration of 0.2 mM H_2_O_2_ (J.T. Baker). Experiments were also performed with different initial H_2_O_2_ concentrations. The concentration of H_2_O_2_ was determined spectrophotometrically using the Ghormley Triiodide method, where I^–^ is oxidized to I_3_^–^ by H_2_O_2_.^48,49^ The absorbance of I_3_^–^ is measured at 360 nm. To make the measurement possible, a volume varying between 80 and 400 μL is extracted from the aqueous UC powder suspension. When H_2_O_2_ is first added to the aqueous suspension, its concentration is high but with time, it decreases due to its reaction with the UC surface. This is the rationale for increasing the sampled volume from 80 to 400 μL over time. Milli-Q water is added to the sample to a total volume of 1.8 mL. 100 μL of 1 M KI and 100 μL of the acetate buffer are added to that 1.8 mL. The acetate buffer contained a mixture of 1 M acetic acid with sodium acetate where a few drops of 4% (wt) (NH_4_)_2_Mo_2_O_7_ that act as a catalyst were added.

## Supporting Information Available

H_2_O_2_ consumption and uranium dissolution as a function of time for 30 mg UC in 25 mL of 10 mM NaHCO_3_ exposed three consecutive times to 0.2 mM H_2_O_2_ (PDF)

## Notes

Vetenskasprådet: 2017-06461.
