# Supplementary material for: On the Stability of Uranium Carbide in Aqueous Solution—Effects of HCO3– and H2O2
Source: ACS Omega. 2021 Sep 10;6(37):24289–95. doi: 10.1021/acsomega.1c04581 (PMC8459425; doi:10.1021/acsomega.1c04581)
Supplement: Supplementary file 1 — ao1c04581_si_001.pdf [file ao1c04581_si_001.pdf]

Supporting Information

On the Stability of Uranium Carbide in Aqueous Solution – Effects of HCO_3^- and H_2O_2

Sawsan El Jamal [a]*, *Mats Johnsson* [b], *Mats Jonsson* [a]

[a] School of Engineering Sciences in Chemistry, Biotechnology and Health, Department of Chemistry, KTH Royal Institute of Technology, SE-100 44, Stockholm, Sweden

[b] Department of Materials and Environmental Chemistry, Stockholm University, SE106 91, Stockholm, Sweden

* Corresponding author: sawsan@kth.se

30 mg of UC powder in 25 mL (10 mM) $NaHCO_3$ was exposed to 0.2 mM H_2O_2 three consecutive times.

After UC synthesis, 30 mg of UC was washed in 10mM $NaHCO_3$ four times to remove the excess carbon left. 25 mL of 10 mM $NaHCO_3$ was then added to the washed UC and this powder suspension was purged with N_2 all along the reaction.

0.2 mM of H_2O_2 was added to the powder suspension. The concentration of H_2O_2 and U(VI) were calculated at different reaction time where two samples were taken at every sampling time. The H_2O_2 was consumed quickly meanwhile the dissolved uranium took longer time to reach a constant concentration.

After the first exposure to H_2O_2, the rest of the solution left was removed and replaced with a new solution of 25 mL 10 mM $NaHCO_3$. 0.2 mM of H_2O_2 was added for a second time on the same 30 mg UC powder. The concentration of H_2O_2 and U(VI) were calculated at the same reaction time as for the first exposure.

The same procedure was repeated for a third time. Figure S1 showcases the calculated concentrations for H_2O_2 and dissolved uranium after 3 consecutive exposures to 0.2 mM of H_2O_2. The concentration of H_2O_2 and U(VI) were very close in those 3 exposures as seen in Figure S1.

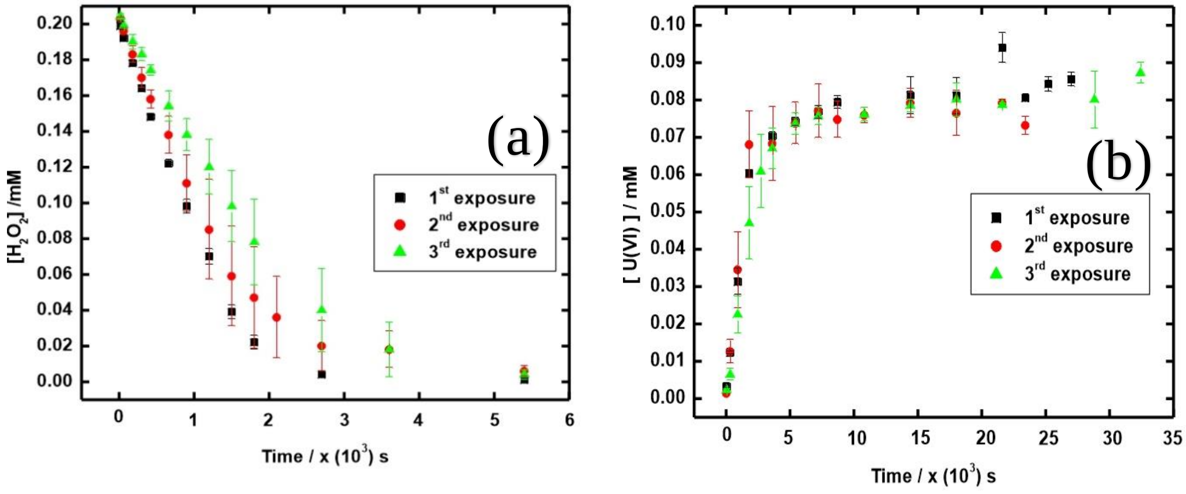

Figure S1. (a) H_2O_2 consumption and (b) Uranium dissolution as a function of time for 30 mg UC in 25 mL of 10 mM $NaHCO_3$ exposed 3 consecutive times to 0.2 mM H_2O_2.
